# Towards a psychological framework on time perception in patients with chronic tinnitus

**DOI:** 10.3389/fnagi.2023.1141903

**Published:** 2023-04-17

**Authors:** Nicolas Dauman

**Affiliations:** ^1^Université de Poitiers, Univ Rennes, Univ Angers, Univ Brest, RPPSY, Poitiers, France; ^2^Maison des Sciences de l’Homme et de la Société, Université de Poitiers – CNRS, Poitiers, France

**Keywords:** tinnitus, time perception, heterogeneity, self-awareness, frustration, enjoyment, hearing aids

## Abstract

Although disabling tinnitus is a chronic auditory phantom sensation, current knowledge on time perception (i.e., subjective time) in sufferers is limited and unsystematic. This theoretical analysis provides a first approach to this topic, highlighting the heterogeneity of time perception in humans as shown in various research areas. This heterogeneity is inherently related to goal attainment. Our immediate perception of time is restricted to present moment and recent past, whereas our sense of time is mostly future-oriented and represented as our past in a mental time line. The heterogeneity of time translates into a tension between anticipated changes one wants to see happen and full commitment that is required to goal attainment. Tinnitus sufferers are intensely aware of this tension in their self-perception. Their most compelling desire is that they no longer perceive tinnitus, but they get closer to this goal only by avoiding to put all their thoughts into it. Our analysis provides new perspectives on acceptance of tinnitus in relation to this time paradox. Building on the Tolerance model and the role of self-awareness in time perception, we contend that the main way for patients to gain long-term self-confidence is to engage in the present moment. Attention to this attitude is obscured in chronic sufferers by worries and ruminations associated with the ongoing presence of tinnitus. We provide arguments that time perception is a social perception, emphasizing the role of rewarding interactions in helping sufferers to overcome the feeling of being prevented from living in the moment. In the course of improvement towards acceptance, different changes in time perception are hypothesized that promote individuals’ disengagement from unattainable goal (i.e., tinnitus suppression). A framework for future research is proposed, which distinguishes individuals’ behaviors and associated emotions in relation to the time paradox.

‘Let anyone try, I will not say to arrest, but to notice or attend to, the *present* moment of time. One of the most baffling experiences occurs. Where is it, this present? It has melted in our grasp, fled ere we could touch it, gone in the instant of becoming.’ William James (1890) *Principles of Psychology*, p. 608.

‘Apart from a few special moments I never really live in the present, I never think of it. But the sickbed does not allow me to escape from the present […] As a patient I live with a useless body in a disconnected present.’ Jan Hendrick Van den Berg (1966) *The Psychology of the Sickbed*, p. 28.

## 1. Introduction

According to the clinical guidelines proposed by Tunkel et al. (2014), the individual reaction to tinnitus can improve over time, without medical intervention, during the first 6 months after the onset of the symptom. This clinical knowledge is important to communicate to patients during this period, in order to reassure them and avoid the multiplication of costly investigations. Thereby, routine examination of the patient’s hearing and education about the mechanisms of tinnitus perception are recommended over potentially invasive interventions. After 6 months the likelihood of spontaneous improvement decreases, although improvement is still possible up to 5 years after the onset of the symptom (*ibid.*). Since most patients enrolled in comparative research report having persistent distress more than 6 months after the onset of their tinnitus, this criterion of duration was advocated in the distinction between acute and chronic (i.e., persistent) tinnitus (*ibid*; see also Cima et al., 2019). The duration is set at 3 months in the guidelines proposed by Mazurek et al. (2022) who emphasized the potential treatment options during this early period, such as cortisone injection therapy and treatment of sudden deafness if associated with tinnitus. After 3 months, the chances of success of these interventions decrease over time and other approaches to tinnitus perception (rather than tinnitus generation) should be considered such as hearing aids or psychological-based therapies. Using the definition of chronic pain as an example, De Ridder et al. (2021) also proposed persistence of tinnitus perception after 3 months be considered chronic. This criterion would also reflect the complex changes over time in brain activity and connectivity, which may contribute to considerable disablement in patients, by turning the tinnitus symptom into tinnitus disorder (*ibid.*). The distinction between acute and chronic tinnitus indicates that the time factor is an important element in recommendable therapeutic strategy and decisions when receiving patients with tinnitus. Equally important in the management of their distress is the *perception of time* by patients themselves, a field of investigation on its own right since the use of metric alone provides little understanding of this dimension. The experience of individuals is the main source of inquiry in this matter, as qualitative studies with tinnitus patients have shown (e.g., Andersson and Edvinsson, 2008; Marks et al., 2019). However, observations related to time in patients with tinnitus are rather limited to date and, furthermore, often do not explicitly refer to time perception as such. The implementation of mindfulness-based approaches to tinnitus is an exception to this remark (Marks et al., 2020). By emphasizing the importance of how patients live the present moment, these approaches further investigate into time perception and lead to a better comprehension of annoyance variability (Dauman and Dauman, 2021). The present theoretical analysis proposes a first systematic approach to this topic in patients with tinnitus and provides a psychological framework for future research.

Time perception (i.e., subjective time) is notoriously an elusive topic which challenges scientific inquiry. Unlike other perceptions, we do not have a sensory organ for the passage of time (Wittmann, 2009) and our sense of time (e.g., duration) is much more unclear than, for instance, our sense of distance by vision (Grondin, 2010). Even though time is intangible, unobservable, and hard to understand, the study of time perception is unmissable as ‘nontemporal behavior does not exist.’ (Hancock and Block, 2012, p. 269). In other words, a scientific inquiry into time perception addresses our interaction with ourselves and ever-changing environment around us. A cornerstone in the inquiry of time perception, which a non-specialist in this field will find useful to keep in mind, is that our perception of time is *heterogeneous*. We do not perceive our past, or conceive our future, in the same way that we experience the moment. Yet these distinct perceptions have strong psychological connections, as James (1890) observed in his pioneering work, this heterogeneity being central to his psychological approach to time (see below, Part 2).

In cognitive psychology, the heterogeneity of time perception shows through the distinction between *prospective* and *retrospective* duration judgments (Block and Zakay, 1997). In prospective judgment, individuals are informed that they will have to give an estimate of the time elapsed during the experiment. Part of their attentional resources is allotted to duration monitoring, which competes with a nontemporal task. As attentional resources are limited, the more complex the task is the less accurate prospective duration judgments are (Zakay and Block, 1997). Duration estimates tend to decrease in length with higher cognitive load (Block et al., 2010), i.e., a shorter time elapses for individuals when stimuli information processing *distracts* them from duration monitoring. In retrospective duration judgment, no information about time is given beforehand and individuals, therefore, have to remember the elapsed duration (Block and Zakay, 1997). In this latter setting, duration estimates tend to increase with higher cognitive load (Block et al., 2010), i.e., individuals consider a longer period has elapsed when stimuli information processing *involved* more cognitive resources. Attention-based (i.e., prospective) and memory-based (i.e., retrospective) processes likely account for the heterogeneity of time perception in experimental settings (see, for a review, Block et al., 2010).

A similar pattern characterizes research in embodied cognition that focuses on time perception in relation to *self-awareness* (e.g., Droit-Volet et al., 2013; Wittmann, 2015; Thönes and Stocker, 2019). Typically, an intensified self-awareness goes with greater awareness of the passage of time, i.e., the feeling that time ‘elongates’ or ‘drags’. For instance, when individuals are asked to make prolonged physical efforts (e.g., holding their breath) they tend to report longer durations (as compared with actual durations), a cognitive bias which reflects the depletion of their body resources due to self-regulation (Vohs and Schmeichel, 2003). Intensified self-awareness also characterizes boredom in meaningless situations, that seem to drag on whenever individuals wish to escape from them and, therefore, induce impulsive behaviors in order to shorten their dissatisfaction (Moynihan et al., 2021). In contrast, the dissipation of self-awareness occurs typically when individuals develop rewarding activities in which they are fully engaged, losing thus their track of time (Csikszentmihalyi, 1990; Wittmann, 2015).

These changes in self-awareness and time perception would not occur by chance, but rather serve adaptive purposes in an evolutionary perspective (Droit-Volet and Meck, 2007; Droit-Volet et al., 2013). This perspective distinguishes engagements in opposite directions according to motivation and goals, with individuals preferring either withdrawal from aversive stimuli or approach to appetizing stimuli (Gable and Harmon-Jones, 2010). Withdrawal motivation (i.e., the desire to increase distance from threatening stimuli) is typically accompanied by the passage of time slowing down (Droit-Volet and Meck, 2007; Gable et al., 2022). The resulting longer perception of time increases the organism’s readiness to act as soon as possible (Gil and Droit-Volet, 2012; Droit-Volet et al., 2013) and, if the aversive situation persists, the distorted perception of time causes the organism to disengage from a situation that overloads the available resources (Gable et al., 2022). In contrast, accelerated passage of time characterizes the desire to get closer to rewarding stimuli, i.e., the approach motivation (Gable et al., 2022). An accelerated sense of time promotes the pursuit of on-going goals as the organism moves closer to their achievement (Gable et al., 2022) and focuses attention exclusively on them (Gable and Harmon-Jones, 2010) to the point of forgetting self-awareness. In sum, the alternation of elongated time perception (with intensified self-awareness) and accelerated time perception (with self-dissipation) would be indicative of the inner resources the organism can afford while pursuing current goals. Time perception would thus inform the self about the *efficiency* with which it engages in goal-directed behavior.

The quotes from James (1890) and Van den Berg (1966) at the beginning of the report might seem contradictory at first glance. Yet they are in line with the above-mentioned approach to self-awareness and time perception. For the former author, the present moment would not exist as such, because our consciousness only apprehends a succession of fleeting impressions (i.e., the present is ‘gone in the instant of becoming’, in James’ words). For the second one, the present moment would be all that remains to the patient who has to face his vulnerability (i.e., ‘a body in a disconnected present’ according to Van den Berg). Actually, when one observes aimlessly passing time, presence to oneself is all one can feel. There is no obstacle against which resources can be tested and driven. On the contrary, the one who suffers and experiences the insufficiency of his resources feels intensively the passage of time while he attempts to resume his usual habits and interactions with his surroundings. The embodied nature of time (Droit-Volet et al., 2013) is highlighted by the constraints of illness.

As Thönes and Stocker (2019) pointed out, the very use of notions such as ‘passage’ and ‘speed’ of subjective time implicitly involves a reference to *space*, since space is necessary to conceive movement. Time being elusive, communication is also facilitated by the use of spatial metaphors that organize temporal events along a symbolic, unidirectional line (e.g., ‘The worst is behind us’ or ‘Thursday is before Saturday’ see Boroditsky, 2000). The widespread metaphor that *time passing is motion* (McGlone and Harding, 1998; Ruscher, 2011) can be rooted to our experience of locomotion in space (Rinaldi et al., 2018). As we move through space, we visually associate the objects we approach with a decrease in the space between those objects and our body, while the distance increases with those we have passed. What is to come in our path (i.e., the future) is in front of us, what is no longer current in our experience (i.e., the past) is behind our body (*ibid.*). Our body’s asymmetrical sensitivity to the stimuli in front of us would also be related to the particular value we attribute to the future, which is associated with our sense of agency (i.e., our ability to act on events in the way we want; Caruso et al., 2008).

The influence of the metaphor ‘time passing is motion’ is reflected in everyday communication about temporal events. A speaker may describe a future event in two ways along a sagittal time line (Boroditsky, 2000). On the one hand, she may describe the passage of time as if she was moving towards the upcoming event (e.g., ‘we are approaching the weekend’). She adopts an *ego-moving* perspective and attributes a stationary position to the event (Boroditsky, 2000; McGlone and Pfiester, 2009; Richmond et al., 2012). On the other hand, the person may describe the passage of time as if the event itself was getting closer to her as time goes by (e.g., ‘The week-end is approaching’). She then adopts a *time-moving* perspective and, importantly, attributes to herself a stationary position (*ibid.*). Although both temporal descriptions are understandable, these perspectives reflect distinct attitudes in the speaker towards the narrated events. Anticipated happiness elicits a greater sense of agency in individuals who tend to use an ego-moving perspective towards rewarding events (Richmond et al., 2012), which is reflected in expressions such as ‘looking forward’ to positive events. On the contrary, a time-moving perspective is associated with a sense of helplessness in individuals who consider future negative events that elicit depressed mood and anxiety. By endorsing a stationary position, they attribute temporal agency to the events they fear will happen. Expressions such as ‘depression descended on me like a darkness’ illustrate this loss of agency in individuals (*ibid.*). In sum, the assignment of temporal agency in communication (either to oneself or to future events) would reflect both our willingness to deal with future events (Ruscher, 2011) and our emotions we associate with their arrival (McGlone and Pfiester, 2009).

In many ways, the preceding remarks suggest that the literature on time perception may provide new perspectives for approaching the individuals’ perception of chronic tinnitus. In particular, the alternative between ego-moving or time-moving perspectives seems relevant to exploring patients’ sense of agency in managing tinnitus over an indefinite period of time (Dauman and Dauman, 2021; Pryce et al., 2023). Individuals enduring tinnitus in their consciousness may feel as if they were forced into a *stationary position*, which they try to counteract by being more active themselves. Intensified self-awareness, coupled with limited resources to cope with the situation, is also likely to exacerbate a time-moving perspective that patients express as a hopeless sense that tinnitus ‘will be there *forever*’ (Colagrosso et al., 2019; Marks et al., 2019). Instead, patients who are more tolerant to tinnitus may put it *in the background* of consciousness (Slater and Terry, 1987; Adams et al., 2010), indicating an ego-moving perspective on the continuous presence of tinnitus. They learn to focus on something else (Pryce and Chilvers, 2018) and continue with their activities (Marks et al., 2022). They anticipate periods of calm beyond a current crisis (Marks et al., 2020) and rely on routines that have proven effective in the past to restore inner balance (Dauman et al., 2017). They remain lucid in their efforts (Pryce et al., 2019) and are more indulgent to themselves than they were at the onset of tinnitus (Marks et al., 2020). These observations suggest that tinnitus may challenge individuals’ time perception and require them to be willing to overcome the feeling of being prevented from *freely living in the moment*. Table 1 provides examples of contrasted time-moving and ego-moving perspectives in patients’ discourses, as illustrated by qualitative studies on chronic tinnitus.

**Table 1:** Ego-moving and time-moving perspectives illustrated from qualitative studies on chronic tinnitus.

TIME-moving perspective (i.e., tinnitus in the front, stationary individual)	EGO-moving perspective (i.e., individual in motion, stationary tinnitus)
‘It fluctuates. ***Today it is bearable, but yesterday it was not. Tomorrow it will return*** more strongly […] One day it’s strong, the next it’s less, ***so you never know***.’ (in Dauman et al., 2017)	‘***Whenever you do*** things you are interested in, you feel much more relaxed. Tinnitus is there, but ***for a while it’s not in the foreground, perhaps even in the background*** (laughing).’ (in Dauman and Dauman, 2021)
‘***When it’s time to go to sleep*** and all that, my head is filled with thoughts about tinnitus.’ (in Andersson and Edvinsson, 2008)	‘***At night***, I still prefer to manage my tinnitus in silence […] ***I know that at other moments during the day***, I will not hear tinnitus for some time.’ (in Dauman et al., 2017)
‘When I talk about it, I think about it or it gets to me, ***it takes a while to go away***. Right now, I can hear it.’ (in Colagrosso et al., 2019)	‘… the sound generator does ***allow you to escape*** […] because it helps you to stop thinking about the tinnitus ***and come outside of it***.’ (in Munir and Pryce, 2020)
‘My tinnitus ***started about 30 years ago*** […] it was sort of devasting. I was thinking ‘I have got ***to live with this loud noise forever***.’ (in Marks et al., 2019)	‘… there is nothing you can do about it so you might as well live with it ***and get on with your life. It can take quite a long time to come to that realization***.’ (in Marks et al., 2022)

The purpose of this theoretical analysis is to investigate *which changes in patients’ perception of time promote the acceptance of tinnitus.*

Part 2. will address a phenomenological approach to time perception, which highlights the heterogeneity of our sense of time. Part 3. will map this approach to time to the Tolerance model (Dauman et al., 2023), showing in particular how temporality can be applied to patients’ frustration in dealing with tinnitus. Changes in time perception will be discussed in more detail in Part 4. with an analysis of the heterogeneity of time (future, past, recent past, and present moment) that we relate to behavior changes in patients. Finally, we will propose a framework for future research on time perception in chronic tinnitus and provide testable hypotheses (discussion, Part 5.).

## 2. Perception of time in William James’ *Principles of Psychology*

In his seminal work, James (1890) provides in-depth insights into time perception that he distinguishes from other perceptions (e.g., hearing, vision) as being our internal perception. In carrying out this theoretical analysis on tinnitus, we will consider in detail three observations inquiring our psychological sense of time.

First, James starts with how our sense of time contrasts by its *narrowness* in comparison to our ability to appraise lengths with our eyes. One only has to look at the window in order to have a feeling of great distances from his own body. In striking contrast with the extended space that one is able to grasp at once through vision, our sense of duration (of time) is very limited: ‘*The units of duration,* James notes, *which the time-sense is able to take in at a single stroke, are groups of a few seconds, and within these units very few subdivisions* […] *can be clearly discerned*.’ (p. 611). Within short durations (i.e., half a minute), our voluntary attention towards the passage of time can be accurate, and even improved by training. However, beyond those instants that have just elapsed, our appraisal of time becomes vague and, a few minutes later, any estimate of duration has completely melted in our grasp. Thenceforth, we only get reliable information about time by looking to our watch. In other words, we cannot rely on our *intuitive* perception of time above short spans of experiencing its passage before our awareness.

This limited range of accurate temporal information usually goes unnoticed. Since our time-sense is ‘*as continuous as any sensation can be*’ (*ibid.*, p. 622), we rather experience time as a stream of sensations and events connected one to another without noticeable disruption. When we are attentive to those stimuli, they seem to unfold before our awareness. That is, a feeling of *pastness* is inherent to our ongoing perception of them. Such a feeling is impossible to overcome in our intuitive sense of time. James even doubted that anyone could seize a single moment of time apart from the stream of his own consciousness (see his citation at the start of this text). Our intuitive sense of time should be referred to as a ‘specious’ present (as suggested by Clay), which continuously appears before our awareness with ‘*a vaguely vanishing backward and forward fringe*’ (p. 613). Not only the specious present contains duration (i.e., our feeling that events are ‘passing’ before us); it also has a *direction* with an ‘earlier’ part and a ‘later’ part. Along this direction, past events are considered to be ‘no more’ in our ongoing perception, and events to come as being ‘not yet’ perceptible.

A second observation results from this careful description. Beyond the restricted boundaries of the specious present, we can only estimate time in a *symbolic* way (i.e., not intuitively). Any notion of duration (i.e., longer or shorter) and direction in time (i.e., before or after) is merely positioned by imagination on a time ‘line’ that is extracted from our sensory experience. Abundant memories associated with some past event lead us to widen our view of it and to remember it with a longer duration than other, less vivid memories. A shorter duration is associated with past events that are not associated to individual engagement and meaning. In sum, our symbolic estimate of time reflects how *we filled in—with our goals—the duration of time* that we consider in retrospect. Future events would follow a similar pattern. Greater durations would be associated (symbolically) with greater diversity and interest, which we anticipate in our engagements with coming activities.

A last observation appears as a paradox in our perception of time, which W. James sums up as follows: ‘*In general, a time filled with varied and interesting experiences seems short in passing, but long as we look back. On the other hand, a track of time empty of experiences seems long in passing, but short in retrospect*.’ (p. 624). In fact, this paradox highlights an alternative in our psychological relationship with time perception. Either we distract ourselves from monitoring the passage of time, by accomplishing valued goals (i.e., ‘*a time filled with varied and interesting experiences*’) or we develop attention to time itself, whenever we fail to engage ourselves with meaningful activities (i.e., ‘*a track of time empty of experiences*’). When we look back to past events, this alternative in our attention manifests to ourselves with a striking contrast. In retrospect, we estimate the duration of these distinct situations (i.e., ‘interesting’ or ‘empty’) in a symbolic way that reflects how much we were *engrossed* with them. Duration in our memory extends with the abundance of events that time contained *for ourselves*. This symbolic relationship would not be fortuitous, but rather certify our continuous search for stimulation. Indeed, lack of commitment and novelty cannot distract ourselves from the ‘*odiousness*’ of the ‘*insipid’* passage of time (p. 626), that is, when time is empty of content and meaning. Full of expectations and waiting for new stimuli, our whole attention decisively stands against such monotony. A pause in music or a halt during a captivating speech make our attitude tangible, as we anticipate for sounds or words to come that would feed *our appetite for rhythm and for change*.

With his psychological analysis of time, W. James provides significant insights into the uninterrupted and meaningless presence of chronic tinnitus in self-perception, when he observes that *‘A day full of excitements, with no pause, is said to pass ‘ere we know it’. On the contrary, a day full of waiting, of unsatisfied desire for change, will seem a small eternity.*’ (p. 626).

## 3. Tolerance model in relation to time perception

**Figure 1:**
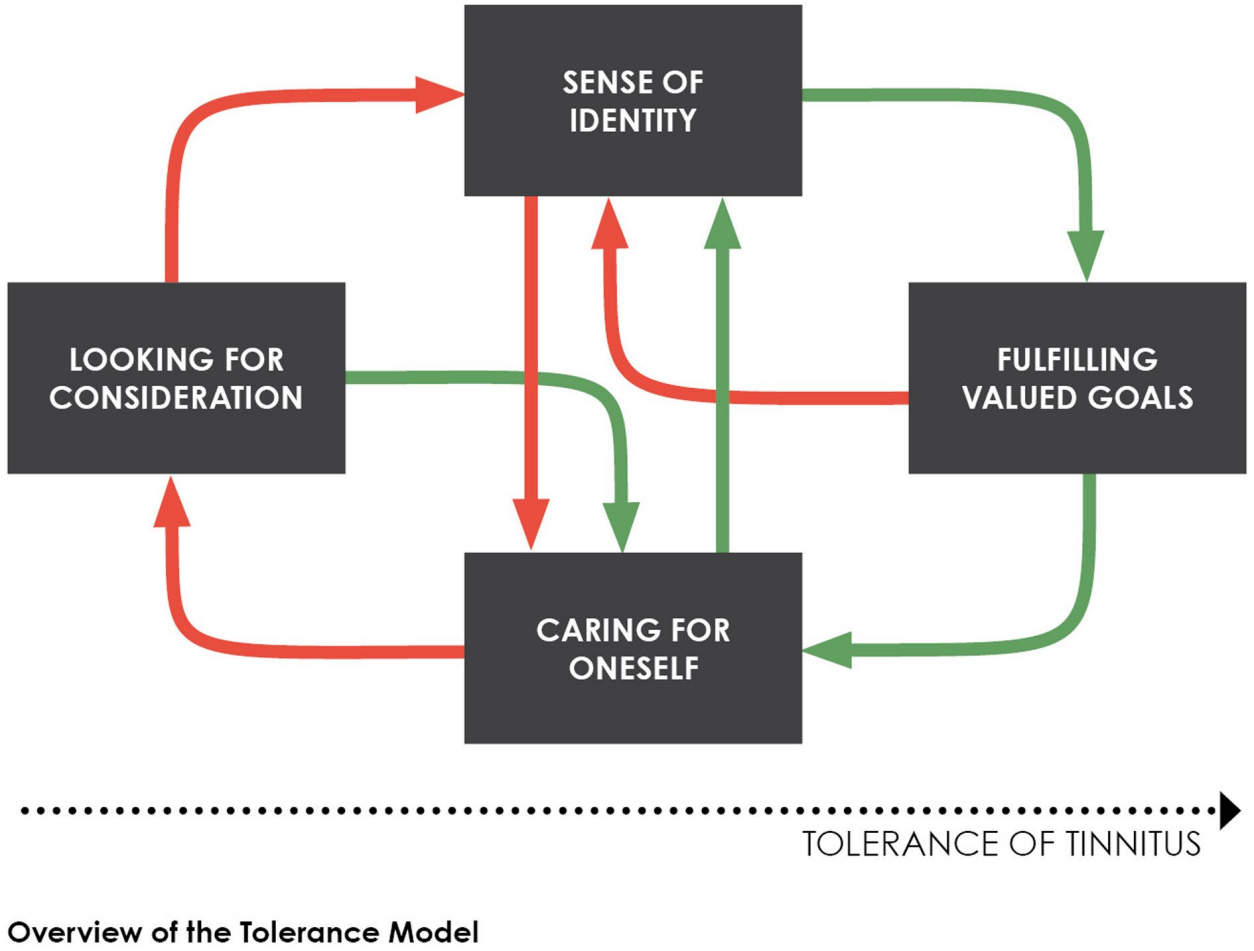
Progress in tinnitus tolerance is supported by the fulfillment of basic psychological needs (green arrows), whereas the frustration of these needs increases the intrusiveness of tinnitus into self-perception (red arrows). In the course of tolerance improvement, initially disrupted sense of identity is restored through meaningful experiences with oneself and others.

Tolerance model is grounded on in-depth interviews with patients who suffer from chronic tinnitus. The model emerged from an inductive, qualitative analysis of their daily experience (Dauman et al., 2017). It has been further elaborated (Dauman and Dauman, 2021) by following a narrative approach to self-perception with tinnitus (Dauman and Erlandsson, 2012; Erlandsson et al., 2020). A recent review of psychological models of tinnitus-related distress defines this model as a humanistic approach to suffering of patients (Dauman et al., 2023). This theoretical analysis will explore further the relevance of time perception in the suggested pathways towards tolerance. Figure 1 provides an overview of the model.

Three basic statements define the model’s approach to tinnitus. They provide a psychological basis to the understanding of the subjective experience of tinnitus over time.

- First, variability in tinnitus intrusiveness, and related distress in the patient, is essential to our psychological understanding of chronic tinnitus (Bouscau-Faure et al., 2003; Dauman et al., 2015). This variability is depicted within the model with green and red arrows that indicate the individual’s *improvement* in tolerance and *worsening* of intrusiveness, respectively. Improvement and intrusiveness are considered within self-perception, for tinnitus is located within the organism’s boundaries (as contrasted with external stimuli, see Dauman et al., 2013). Furthermore, the model posits that the individual’s goal-directed behaviors can modulate self-perception and intrusiveness of tinnitus.
- Second, the model suggests that fluctuation in intrusiveness reflects the amount of *frustration* the individual encounters *in pursuing their goals* (Dauman et al., 2017; Dauman and Dauman, 2021). Frustration is the feeling elicited by the inability to change a situation as one would wish. The greater the feeling of frustration in the individual, the worse the intrusiveness of tinnitus in self-perception. Illustrative of persistent frustration is the inability to get rid of tinnitus definitively. The model defines frustration *management* as a core variable in dealing with chronic tinnitus. This core variable is bidirectional. On the one hand, goals-fulfillment is accompanied with a softening of the intrusiveness of tinnitus and the dissipation of niggling self-awareness. On the other hand, the thwarting of goals-pursuit is associated with worsening in intrusiveness. Many obstacles associated with tinnitus may interfere with the individual’s pursuit of their goals (e.g., hearing difficulties, noise sensitivity, lack of understanding by others, interpersonal conflicts). Interference with goals-directed behaviors fuels rumination in the sufferer about having tinnitus (Trevis et al., 2016; see also Nolen-Hoeksema et al., 2008). In turn, rumination hinders the individual’s sense of smooth engagement with ongoing activities that distract him from the intrusiveness of tinnitus.
- Third, disabling tinnitus jeopardizes the patient’s *sense of identity* in dealing with a burden that cannot be removed from self-perception (Dauman et al., 2023). The model considers tinnitus in relation to psycho-social factors that are inherent to living with chronic illness. These factors have been identified in patients’ self-narratives with tinnitus (see, e.g., Marks et al., 2019, 2020; Erlandsson et al., 2020; Pryce et al., 2023). In the model, they are labeled with plain words that patients can understand at once: (1) *looking for consideration*, (2) *caring for oneself* and (3) *fulfilling valued goals*. These factors resonate with basic psychological needs that contribute to personal growth and long-term well-being (i.e., relatedness, autonomy, and competence, see Reis et al., 2000). They are also in line with a biopsychosocial perspective on tinnitus-related distress that has been recently promoted in the literature (Erlandsson et al., 2020; Marks et al., 2020). The model emphasizes that such factors involve interpersonal resources (e.g., within interactions with the health professionals, the patient’s partner, their relatives and friends, their workmates). Suffering patients look for consideration from others, and health professionals’ consideration is instrumental in dealing with tinnitus. Similarly, the individual can fulfill valued goals on his own, but goals-fulfillment by others also provides useful distraction from tinnitus. In accordance with this perspective, patients’ sense of identity is strengthened when their needs are *nurtured* by supporting interactions with their social surrounding. On the contrary, confusion and worsening of intrusiveness in self-perception results from the *deprivation* of these basic needs (Erlandsson et al., 2020; see also Reis et al., 2000).

Tolerance model of tinnitus was elaborated with no reference to James (1890) psychological analysis on time perception. Yet, it is noticeable that these frameworks share common perspectives on time and tinnitus perception.

In particular, the *alternative* that is core to James’ approach to time perception echoes with the current analysis of intrusiveness of tinnitus. Either we distract ourselves from monitoring the passage of time, James observes, by pursuing valued and enticing goals, or we grow attentive to time itself when we fail to engage in meaningful situations. In the same way, the Tolerance model posits that goals-fulfillment in patients is accompanied with the dissipation of both niggling self-awareness and the monitoring of tinnitus (see for details Dauman and Dauman, 2021). Conversely, patients grow attentive to the salience of tinnitus when they are not engaged in activities they can pay attention to (see also Marks et al., 2020).

Both analyses explicitly relate this alternative in time and tinnitus perception (i.e., either ‘ongoing distraction’ or ‘growing attention’) to the individual’s *wish* for novelty. According to James (1890), we grow attentive to time when we confront ourselves with ‘*a day full of waiting, of unsatisfied desire for change’* (p. 626). Tolerance model endorses a similar viewpoint on the fluctuation of salience, by suggesting that intrusiveness of tinnitus worsens with increased *frustration* in the patient. In both analyses, the role of goals-fulfillment is equally important (i.e., the feeling of achievement in one’s pursuit of valued goals). The passage of time goes unnoticed, in James’ observation, during ‘*a day full of excitements, with no pause*’ (*ibid.*, p. 626). The same way, tinnitus may go unnoticed the moment before patients cease performing meaningful activities (i.e., tinnitus salience increases with the cessation of activity, see Adams et al., 2010; Dauman et al., 2017).

Tolerance model shares a last dimension with James (1890) analysis of time perception, as both distinguish *four alternatives* in the individual experience of tinnitus and time. Perception of time is both intuitive and symbolic, for we compensate the narrow range of our ongoing perception of time with representations of events (i.e., obvious past and future) on a mental time line. Close attention to the passage of time can lead to skepticism about our ability to attend to a discrete moment of time that deserves to be called ‘the present’ moment (*ibid.*, p. 608). Our intuitive perception of time is rather that of continuous pastness of present stimuli (i.e., ‘recent past’, that is associated with movements and changes in the stimuli). James (1890) thus distinguishes four alternatives in time perception, depending on the kind of activity one is involved in. On the one hand, three of these experiences are related to the *stationary* position of an observer of the passage of time. The experiences of the obvious past and future (i.e., both symbolic) and that of the recent past (i.e., intuitive) are related to such stationary position, where the observer *stop moving in order to pay attention to time*. On the other hand, the experience of the present moment can *also* be associated with *a moving position in the individual*, which then attenuates awareness of time. The alternative in time perception involves such distinction of four experiences that are elicited according to whether the perceiver adopts a moving (i.e., present moment) or a stationary position (i.e., obvious past, recent past, and future). Similarly, the Tolerance model distinguishes four behavior circuits and individual attitudes toward tinnitus in self-perception (Dauman and Dauman, 2021). Table 2 sums up the correspondences between these circuits and the experiences of time according to James (1890).

**Table 2:** Theoretical correspondences between time perception (in James, 1890) and tinnitus perception (in Dauman and Dauman, 2021).

Time perception	Tinnitus perception	Time perception in patients with chronic tinnitus
Future (symbolic and stationary perception)	Circuit 1. Helplessness and relapse	Frosty future: anxiety and disrupted self-regulation
Obvious past (symbolic and stationary perception)	Circuit 2. Control of intrusiveness	Mourning past: depression and grief from accumulated losses
Recent past (intuitive and stationary perception)	Circuit 3. Detachment from activities	Vanishing past: boredom and interrupted engagements with others
Present moment (intuitive and moving perception)	Circuit 4. Self-induced relief (optimal experience, i.e., flow)	Enticing present: enjoyment from collapsed time monitoring

## 4. Changes in time perception in patients with chronic tinnitus

Behavior circuits in the Tolerance model are depicted from the higher levels of distress to gradual improvements in tinnitus tolerance. In accordance with this view, the heterogeneity of time perception in patients will be addressed in the following order: future (circuit 1), past (circuit 2), recent past (circuit 3) and present moment (circuit 4). Attention to the moment is the *last* experience in sufferers, as enjoyment is obscured by worries, ruminations and frustrations associated with the ongoing presence of tinnitus in their self-perception. Each perception of time will be first illustrated by patients’ observation on tinnitus, as this theoretical analysis is grounded on qualitative studies.

Broadly, changes in time perception that are hypothesized here concern the *direction*, the *passage* and the *value* of time for tinnitus patients. A major change that would accompany the individuals’ gradual tolerance is a shift from a *time*-moving to an *ego*-moving perspective about tinnitus. The felt passage of time would also be altered in the course of improvement, being accelerated by anxiety and slowed down by depression and boredom. Notably, the acceptance of tinnitus is best characterized by individuals’ loss of *track of time* when engaged in rewarding activities. Eventually, the resignation of distressed patients before the perceived accumulation of time (i.e., tinnitus is ‘there forever’) would turn to a *valuation* of the time shared with others, and greater attention individuals pay to themselves in the present moment.

### 4.1. Frosty future: Anxiety and disrupted self-regulation

“*At first, you feel really overwhelmed by tinnitus. So you think you will remain this way all your life. First because the ENT specialist I met told me so. He said: ‘Your hair cells are destroyed, they will not grow again. So, you will have tinnitus all your life’. […] When a physician tells you that, it’s crazy.*” (Female participant, 63 years old. Tinnitus duration: 13 years).

Patients with tinnitus commonly fear that intrusiveness will worsen over time and become out of their control (Stouffer and Tyler, 1990; Dauman and Bouscau-Faure, 2005; Davis and Morgan, 2008; Dauman et al., 2017; Marks et al., 2019). It is typical for distressed patients to worry about their future (Andersson and Edvinsson, 2008), lifestyle (Tyler and Baker, 1983) and quality of life with tinnitus (Watts et al., 2018). The inherent uncertainty of the future (Caruso et al., 2008) increases worries that are associated with the unanticipated onset of tinnitus (Marks et al., 2019).

**Figure 2:**
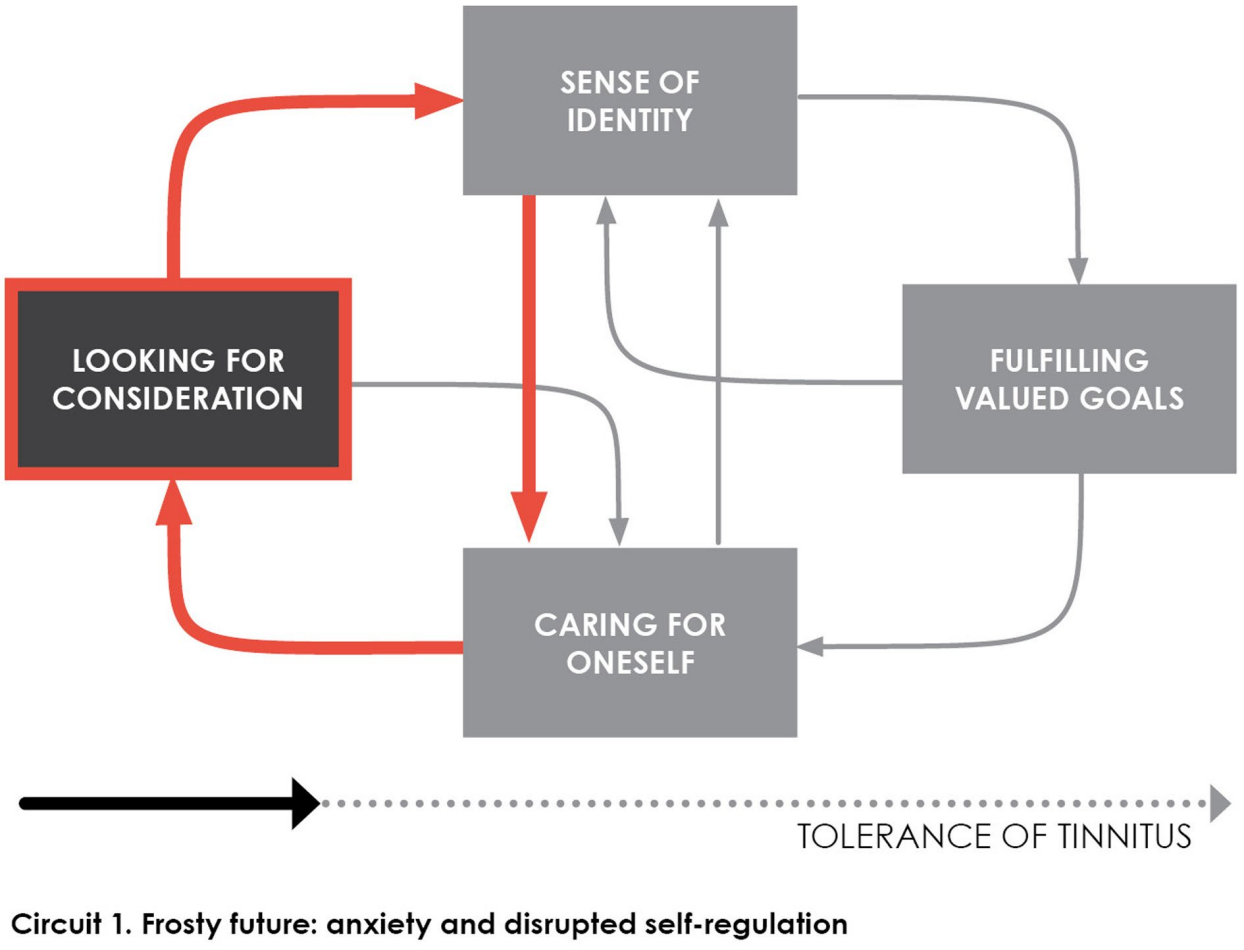
The individual’s experience is dominated by anxiety about a dead-end future when no one helps him to step back from the intrusion of tinnitus. A vicious cycle develops that impoverishes the individual’s sense of identity due to the disruption of self-regulation and the inability to find ways to take care of oneself.

Tolerance model posits that *consideration* for patients is key in their perception of tinnitus over time, and has crucial impact upon their *self-regulation* (Dauman and Dauman, 2021). As depicted in Figure 2, lack of consideration (red arrows) deprives them of reliable perspective about tinnitus, for individuals being overwhelmed by tinnitus can only appraise the course of tinnitus through their present sense. With no perspective of positive evolution emerges increased anxiety and disrupted self-regulation.

Human beings have a normal future orientation of time perception, which is unquestioned in healthy individuals (Davies, 1997; Holman and Silver, 1998). Fulfilments of personal and socially valued goals, associated with a sense of body ownership, are usually taken-for-granted as part of expectations about future in such individuals (Bury, 1982). In contrast, chronic illness can dramatically challenge these beliefs, throwing individuals into unanticipated restrictions and dependency to others in their daily lives. Such a change is a biographical disruption (*ibid.*).

In the case of tinnitus, health professionals have significant influence over patients’ sense of agency in dealing with their symptom. In particular, an exclusively cure-focused discourse is counterproductive for sufferers who wander for months searching for relief through the suppression of tinnitus (Marks et al., 2019). Greater uncertainty also arises from the help-seeking process itself, with patients having to wait for further examinations after consultation with their GP (Pryce et al., 2023). In addition, patients are confused in thinking about their future as they widely have to discover by themselves ways of coping with tinnitus that are seldom explicitly articulated to them (*ibid*).

The symbolic association between the perspective over one’s future and the diversity of activities that one will undertake (James, 1890) suggests that monotony of tinnitus generates strong protest in patients who suffer from it. This hypothesis is in line with high levels of anxiety in distressed patients (Hesser and Andersson, 2009) who complain that tinnitus spoils every goal they pursue (Dauman et al., 2017; Watts et al., 2018). Anxiety results from endless struggle with tinnitus, which always returns in the forefront of awareness whenever patients are attempting to avoid it (Hesser et al., 2009). Patients’ protest against an impoverished future as a consequence of tinnitus is characterized by the narrowing of their scope on self-perception. Selective attention (McKenna et al., 2014) and catastrophizing (Cima et al., 2011) are established cognitive biases that accompany the patients’ attempt to *reject* tinnitus out of themselves. High motivation in pursuing this goal suggests that, in distressed patients, the passage of time is speeded up (Gable and Harmon-Jones, 2010). Their compulsive search for a cure becomes disconnected from the clock-and-calendar time of their surrounding (see Hellström, 2001). Suffering individuals thus multiply consultations with physicians (Brüggemann et al., 2016) and feel their time is being wasted with no effective intervention on tinnitus (Dauman and Dauman, 2021).

Psychological research suggested that individuals’ ability to imagine their future relies on how specific (i.e., contextualized) their autobiographical memories are (Williams et al., 1996). Specific memories (with places, relationships and personal feelings) would facilitate concrete and achievable goals in people taking advantage of their remembered experiences. Lacking reliable perspective over their future, suicidal patients typically have overgeneralized memories (i.e., vague and restricted) upon which they cannot base effective goals-pursuit. Similar pattern of impoverished memories was reported in patients with chronic tinnitus (Andersson et al., 2003) and chronic pain (Quenstedt et al., 2021). Both groups of patients with chronic illness would have difficulties in imagining their future in a practical way, because of present concerns with intrusiveness (Meyer et al., 2015; Quenstedt et al., 2021). Ceaseless efforts to deal with intrusiveness of tinnitus (Marks et al., 2019) would also narrow time perspective in disabled patients, in relation to self-depletion of energy and inability to devote attention to distant future goals (Vohs and Schmeichel, 2003).

A *time-moving perspective* (McGlone and Harding, 1998; Richmond et al., 2012) is characteristic of distressed patients in general. They suffer intrusiveness in a passive way, as if they were stuck in a *stationary* position with time accumulating over them. Although they devote considerable energy in distractive activities, they feel enable to outrun tinnitus which always stands over their consciousness. Risk of exhaustion is high in a struggle that is defeated by the endless returns of tinnitus in self-perception (Slater and Terry, 1987; Hébert et al., 2012). According to the present analysis, high motivation to withdraw from tinnitus cannot be sustained by patients in the long term. Persistent anxiety and fruitless efforts in diverting attention away from the internal noise would rather result in depressive mood, with noticeable changes in time perception.

### 4.2. Mourning past: Depression and grief from accumulated losses

“*My brain compensates so that I don’t overly busy myself with tinnitus, but after a while it no longer succeeds in doing so. In the past, this was the moment when, having gone too far, I used to get depressed. But now that I know myself better, I try to slow down and pause. I am more withdrawn over myself and have fewer contacts with people. This is my limit for being able to deal with tinnitus.*” (Female participant, 60 years old. Tinnitus duration: 11 years)

Depression is widely documented in patients with tinnitus (Langguth et al., 2011; McKenna et al., 2014; Trevis et al., 2016) and rumination about one’s life before the onset of tinnitus is typical in sufferers (Trevis et al., 2016; Erlandsson et al., 2020). In the wake of disabling tinnitus, many losses have been identified that account for changes in self-perception that individuals find hard to overcome. Part of these losses concerns intimate dimensions of self, such as free attention (Marks et al., 2019) and peace of mind (Pryce and Chilvers, 2018), relaxation doing nothing special (Dauman et al., 2017) and being able to fall easily asleep (Munir and Pryce, 2020). Other losses relate to social life and translate as one’s isolation from relationships with others (Dauman and Dauman, 2021) and reluctance to become a burden for close relatives (Marks et al., 2019).

**Figure 3:**
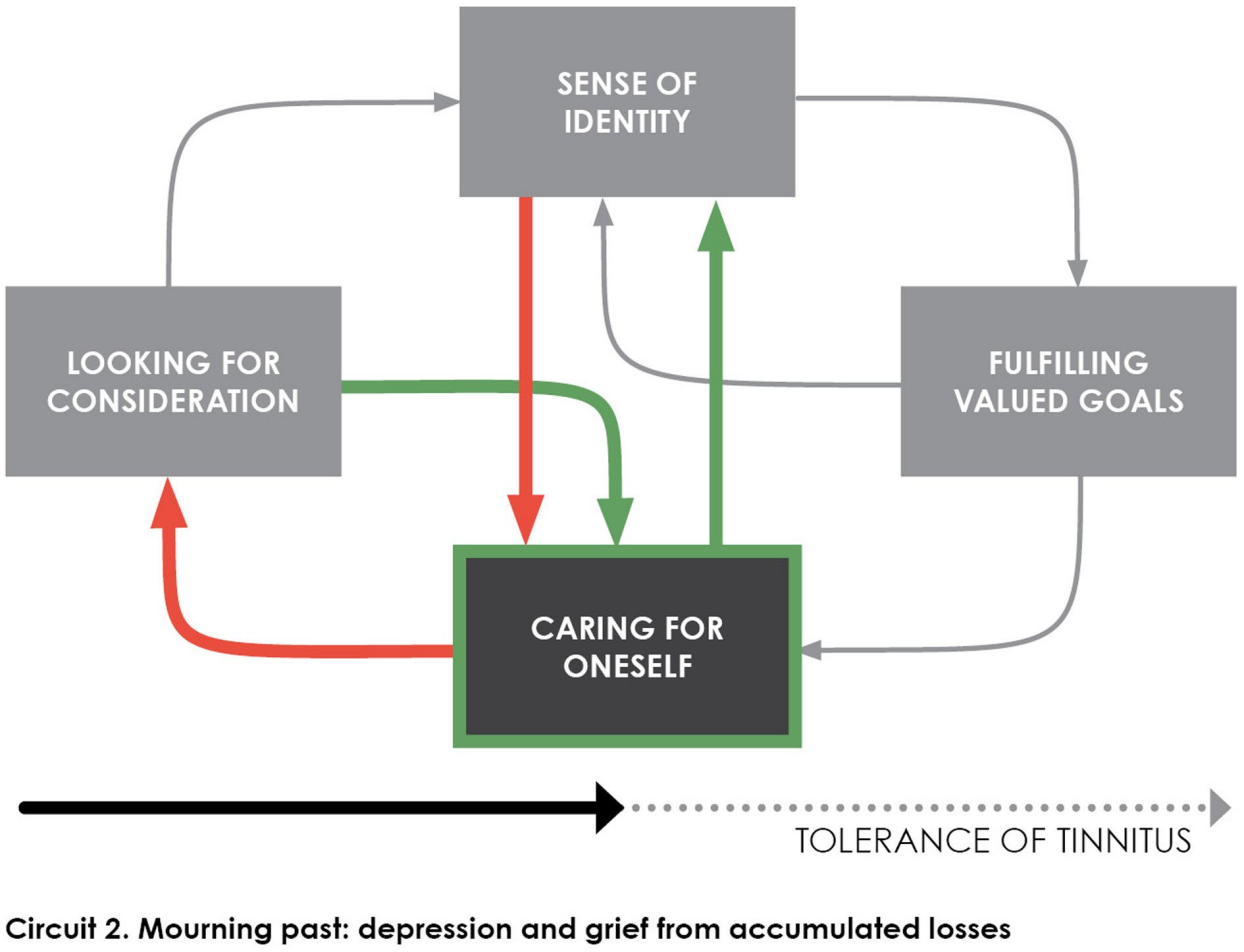
The individual refocuses on himself in a depressive withdrawal when he realizes that his resources are limited in face of continuous presence of tinnitus. Consideration from health professionals and his entourage allows this disengagement from the unattainable goal of suppressing tinnitus in which the individual unwisely consumed his resources.

Tolerance model posits that *caring for oneself* replaces, over time, patients’ initial striving for a cure that would free them from intrusiveness (i.e., rejection of tinnitus). Consideration from others, including professionals, eases this shift in searching for relief (Marks et al., 2022), but even individuals who gained improved tolerance remain interested in an accessible treatment that would eventually suppress tinnitus (Adams et al., 2010; Marks et al., 2019; Pryce et al., 2019). This dual attitude (i.e., looking for relief from outside and inside) is depicted in Figure 3 with green arrows and red arrows in opposite direction. If patients find ways to care for themselves they also strengthen their sense of identity, but feeling of helpless in doing so drives them easily back to external assistance searching (see Ryan and Deci, 2000).

Ruminating on one’s past is characteristic of self-narratives of patients who feel unable to cope with chronic tinnitus (Erlandsson et al., 2020). Suffering patients also narrate how their life goals remain unattainable in their views (*ibid*), which is consistent with a sense of passivity and a *time-moving perspective* (Richmond et al., 2012). Similar to the anxiety-driven rejection of tinnitus, patients’ resignation is associated with a *stationary* perception of themselves in front of the relentless presence of tinnitus. Although there is evidence to suggest that depression is associated with maladaptive behavior in tinnitus patients (see, for review, Trevis et al., 2018), arguments exist that support an alternative interpretation of depression as being an adaptation to unpropitious situations (Nesse, 2000). This alternative view is enlightened by further analysis in the perception of time.

Passage of time is typically slowed down in the awareness of patients with depression (Thönes and Oberfeld, 2015). Depressed individuals report that time seems to ‘drag’ for them, a feeling that is associated with psychomotor retardation (Blewett, 1992). With reference to Nesse (2000), the present analysis suggests that such alteration in time perception is adaptive in depressed patients who must deal with disabling tinnitus. Indeed, the slowing down of the passage of time is associated with increased self-awareness, an aversive experience that is precluded by the anxiety-driven rejection of tinnitus in oneself. With lack of energy available for distraction, depressive self-focused perception (Pyszczynski and Greenberg, 1987) makes it obvious to individuals that they must manage *limited* resources in dealing with chronic (i.e., unlimited) tinnitus. Therefore, a slower perception of time in depressed mood contributes to patients’ conservation of resources, which limits the sense of helplessness in front of accumulated stressors (Höbfoll, 1989). Following this realization, patients adopt a more regular lifestyle to avoid wasting energy and exposing themselves to inconsistent social demands (Dauman et al., 2017). Self-focused perception also makes them realize how counterproductive their struggle with tinnitus is, as it merely increases their frustration at not being able to get rid of it (Dauman and Dauman, 2021).

When individuals exhaust themselves in pursuing unreachable goals, the inhibition of activity—which is characteristic of depression, whatever its cause—is more adaptive than the opposite (Nesse, 2000). Constant distraction from tinnitus (Hesser et al., 2009) and attempt to get rid of it definitively (Dauman et al., 2017) can be seen as such unattainable goals. In line with this view, disengagement from the restless pursuit of these goals (see Wrosch et al., 2003) enables individuals’ progress toward acceptance of tinnitus (McKenna et al., 2018; Dauman and Dauman, 2021).

The inhibition of activity also contributes to self-preservation in the face of a suicide risk, as committing suicide was reported to be an attempt to escape from unbearable self-awareness (Baumeister, 1990). Higher prevalence of suicidal ideation was found in patients with tinnitus as compared to general population (e.g., 13 vs. 9.8%, respectively, in Aazh and Moore, 2018). However, prevalence of suicidal behavior in tinnitus patients is much less (around 0.25% in Cheng et al., 2023), which indicates that ideation about self-harm most of the time does not translate into suicide attempts. In agreement with this statement, Martz et al. (2018) found no significant correlation between depression in veterans diagnosed with tinnitus and increased likelihood of death caused by suicide. Quite the opposite, they reported a lower risk of suicide in this later group than in veterans with no tinnitus. Although counterintuitive, these observations are in line with an adaptive function of depression associated with the slowing down of the passage of time and the inhibition of suicide risk. Self-focused perception and rumination about the past may pave the way to greater awareness into one’s resources and patterns of behaviors that can restore a sense of agency in patients (Dauman and Dauman, 2021). A slower time awareness would be a determining factor in this process of personal growth.

### 4.3. Vanishing past: Boredom and interrupted engagement with others

“*With my hearing aids I try to rise above the tinnitus. However, this is not always possible. When I am particularly busy, I manage not to listen to it. Nevertheless, when I am inactive or at table with many people talking at the same time, I get the feeling that tinnitus is worse. […] Shortly after hanging up the phone* [with the psychologist]*, the whistling will be louder. I will find myself back to a non-speaking situation and have the feeling that my head is going to explode. Afterwards, I’ll move, drink a glass of water, and will feel better. I will carry out another activity and once again forget to listen to my noise. And I will hear it again, perhaps, half an hour later.*” (Male participant, 62 years old. Tinnitus duration: 16 years)

Interest in how patients live the moment expanded with the implementation of mindfulness-based approaches (e.g., McKenna et al., 2018; Marks et al., 2020), attentional models of tinnitus-induced annoyance (e.g., Trevis et al., 2016) and experience sampling methods with smartphone that report moment-to-moment variability in annoyance (e.g., Schlee et al., 2016). In regard to time perception, a hallmark feature of tinnitus is the patients’ sensitivity to interruption in their goal-directed behavior (Trevis et al., 2016; Dauman and Dauman, 2021) and to changes in their auditory surrounding (Hébert et al., 2013; Pan et al., 2015; Vielsmeier et al., 2016). The interference of tinnitus with activities is a major threat to quality of life (Watts et al., 2018) and, as suggested in pain by Eccleston and Crombez (1999), the recovery of chronic interruption is significant in the burden of tinnitus. According to James (1890) analysis, these observations concern our perception of an ever-changing environment that is associated with a sense of pastness. Observing vanishing past, patients usually have the feeling that they are prevented from freely engaging with the moment.

**Figure 4:**
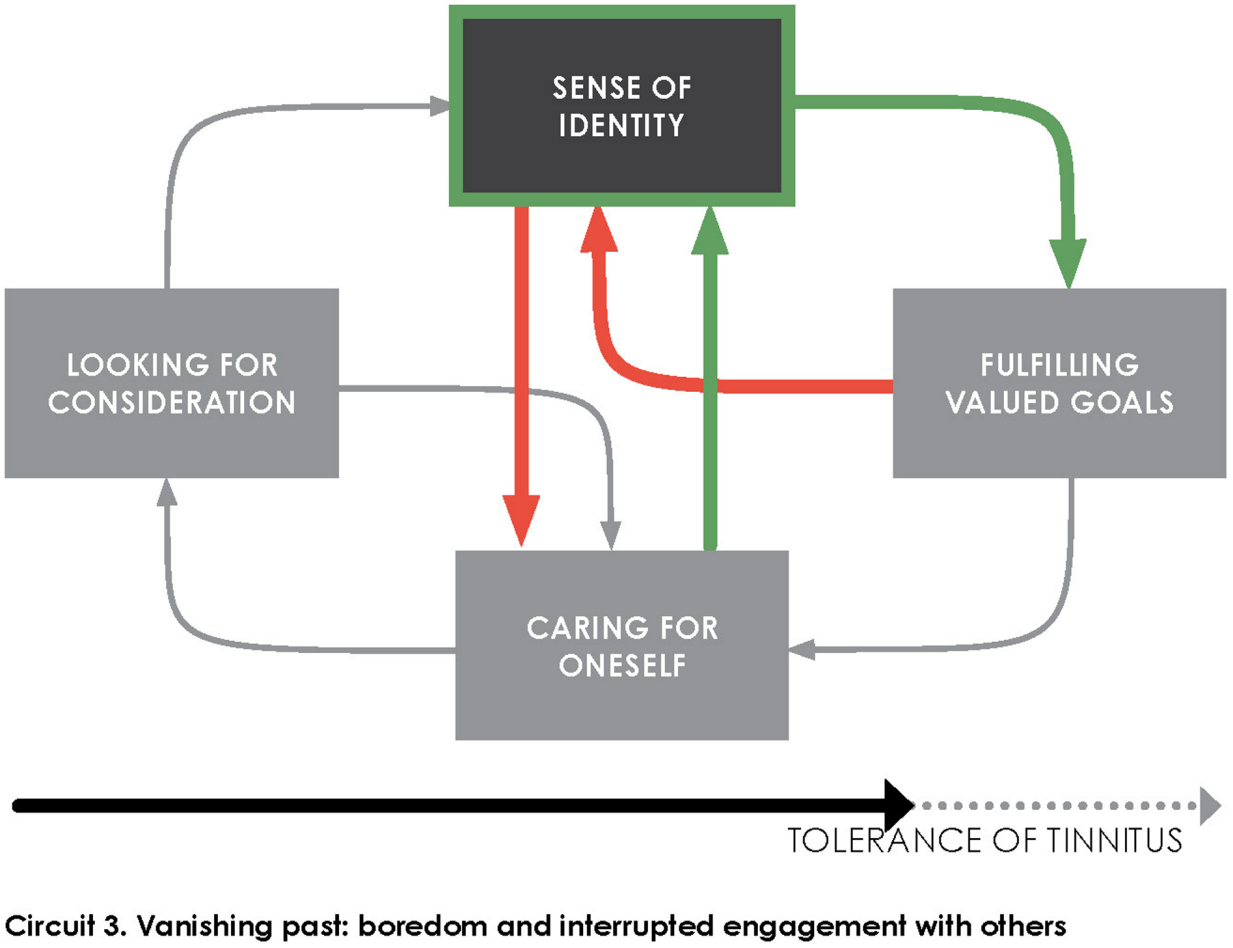
The individual finds momentary relief in rewarding interactions, but often experiences boredom when interrupted by rapid return of tinnitus into their consciousness. Personal routines, past experiences of transient worsening, and cultivated self-indulgence help to put the intrusiveness of tinnitus into perspective.

Tolerance model posits that *pursuing valued goals* is key in the patients’ improvement towards tinnitus acceptance, in line with consistent observations in the literature (Slater and Terry, 1987; Adams et al., 2010; Pryce and Chilvers, 2018; Colagrosso et al., 2019; Dauman and Dauman, 2021; Marks et al., 2022; Pryce et al., 2023). However, chronic interruption in the pursuit of patients’ goals triggers in them a sense of *niggling self-awareness* (Dauman and Dauman, 2021) which fuels rumination (self-focused thoughts) and a sense of discouragement. Repeated shift from goal-directed behavior to self-focused attention is depicted in Figure 4 with green arrows and red arrows in opposite direction. Relief that patients experience in goal-fulfillment may be short, tinnitus returning rapidly in their consciousness when they are interrupted or finished in doing so.

It is well-established that quiet environments can enhance the salience of tinnitus in patients who report that tinnitus suddenly ‘shows up’ (Colagrosso et al., 2019), is worsened (Pan et al., 2015) or returns in the forefront of consciousness (Dauman et al., 2017; Beukes et al., 2018). Likewise, increased levels of noise may worsen salience, as well as the contrast between the noise by which individuals were surrounded just before and their current (softer) acoustic environment (Pan et al., 2015). Auditory sensitivity in patients with tinnitus (Hébert et al., 2013) translates into greater difficulties to comprehend speech in noisy situations and filtering out irrelevant sounds (Vielsmeier et al., 2016; Ivansic et al., 2017). Sensitivity to change in attentional focus is also illustrated by increased salience following termination of engrossing activities (Dauman et al., 2017) and, in many patients, just talking about tinnitus with others gets them notice it vividly (Pryce and Chilvers, 2018; Colagrosso et al., 2019; Marks et al., 2022). These observations suggest that patients easily feel expelled from living the ongoing moment. As Eccleston and Crombez (1999) suggested in chronic pain, such interruption may threaten individuals’ need for meaning, as impoverished interactions with others inevitably follow.

According to the literature on boredom, individuals strive to get involved with meaningful situations providing them with an optimal amount of information on a regular basis (Eastwood et al., 2012; Zakay, 2014; Moynihan et al., 2021). Impoverished interactions induced by the monotony and meaningless presence of tinnitus, with its repetitive returns into consciousness, are likely to induce boredom in patients. Boredom is the feeling of ‘wanting but not being able to engage in satisfying activity’ (Eastwood et al., 2012, p. 482). Like depression, this emotion is associated with a slowing of passage of time (Zakay, 2014) and the feeling that boring situations last longer (Elpidorou, 2014). Passage of time is felt intensely in boredom since much attentional resources are allotted to prospective timing (Zakay, 2014). This situation is experienced by patients who are disrupted in their activities and monitor the enduring return of tinnitus in consciousness.

When people are bored they feel trapped in an unwanted situation and monitor their failure to find meaning in the unpleasant moment (Eastwood et al., 2012). Boredom threatens their sense of agency because they have no purpose to engage with (*ibid.*). Yet boredom is useful also, since it informs the self—though distorted time perception—of the pressing need to find alternative goals that will provide more satisfaction (Elpidorou, 2014). The irritation that accompanies boredom prompts individuals to leave the unsatisfying moment (*ibid.*). In tinnitus, boredom might help individuals sustain their routines despite the dull presence of tinnitus (Slater and Terry, 1987; Dauman et al., 2017). As repetitive patterns of highly predictable and relaxing actions, routines help individuals shorten the perceived duration of time (Avni-Babad and Ritov, 2003). In particular, routines diminish the complexity of one’s surrounding and resume attention to familiar sequences of events. Walking outdoors is such a helpful routine in tinnitus (Slater and Terry, 1987), enabling patients to break physical immobility that worsens depressive mood (Holgers et al., 2000).

Mindfulness provides new insights on the changes in time perception in patients who develop acceptance of tinnitus (see Marks et al., 2020). In particular, it promotes an alternative attitude towards tinnitus that is neither distraction nor routines, by purposefully allowing the noxious presence of tinnitus in self-perception (*ibid.*). Through regular practice, patients learn to rid themselves of both resignation (induced by a stationary position in front of ongoing tinnitus) and exhaustion (trying to be ahead of tinnitus with constant distraction). Rather than being tensed up on tinnitus, they broaden their attention to *simultaneous* stimuli they carefully experience (e.g., background sounds, others’ voices, one’s own breathing). Growing attention to the flow of consciousness is associated with patients’ wish to *take time* for themselves (*ibid.*), in clear contrast with their previous reluctance not to be constantly busy. Patients realize that worsened intrusiveness *pass on* like other phenomena, which softens the fear of being trapped by tinnitus. Essential to this renewed experience with the moment in patients is the absence of judgment on oneself and tinnitus (*ibid.*).

An *ego-moving perspective* characterizes improvement in acceptance of tinnitus, with a lower and a higher level of changes in patients’ perception of time. The former level relies on the dynamics of boredom with attempts at preserving the self from meaning threat by means of distraction and routines (Avni-Babad and Ritov, 2003; Moynihan et al., 2021). The latter level cultivates indulgence with oneself (i.e., self-compassion, see O’Dea et al., 2022) which enables to overcome boredom with greater efficacy in the long term (Marks et al., 2020). Mindfulness helps patients broadening their sense of self and inner resources to put transient intrusiveness in perspective. Both levels foster the individuals’ attention in the moment and contribute to the *embodiment* of tinnitus as being part of oneself (Munir and Pryce, 2020).

### 4.4. Enticing present: Enjoyment from collapsed time monitoring

“*Manual work is the most efficient. Even in silence I manage not to hear my tinnitus then. When I am very busy manually, that is good, I am settled down, my work is perfect and then I don’t hear it […] This afternoon, I was tinkering on a motorbike quietly in my workshop. Without any noise, just the sound of the keys and my full focus. I was concentrating on what I had to do to make it work well and then, for two or three hours, I did not hear my tinnitus.*” (Male participant, 56 years old. Tinnitus duration: 12 years)

Acknowledging the inherent search for meaning in individuals, the concept of *acceptance* of tinnitus was promoted in the literature (see Hesser et al., 2015), differing from a more traditional view relating patients’ relief to habituation to the noise (see, for review, Dauman et al., 2013). This change in the approach of tinnitus progress was emphasized by Hesser et al. (2015, p. 176) who indicate that acceptance is ‘used as a way [for patients] to persist or engage in effective actions to pursue valued goals in life’. The importance of having attainable goals was earlier promoted by Slater and Terry (1987) who noted how instrumental absorbing activities can be, advising patients to cultivate consistency in pursuing well-mastered activities so helpful for them. Importantly, the acceptance of tinnitus involves changes in how individuals spend their time and resources in meaningful goals—instead of struggling against the presence of tinnitus.

**Figure 5:**
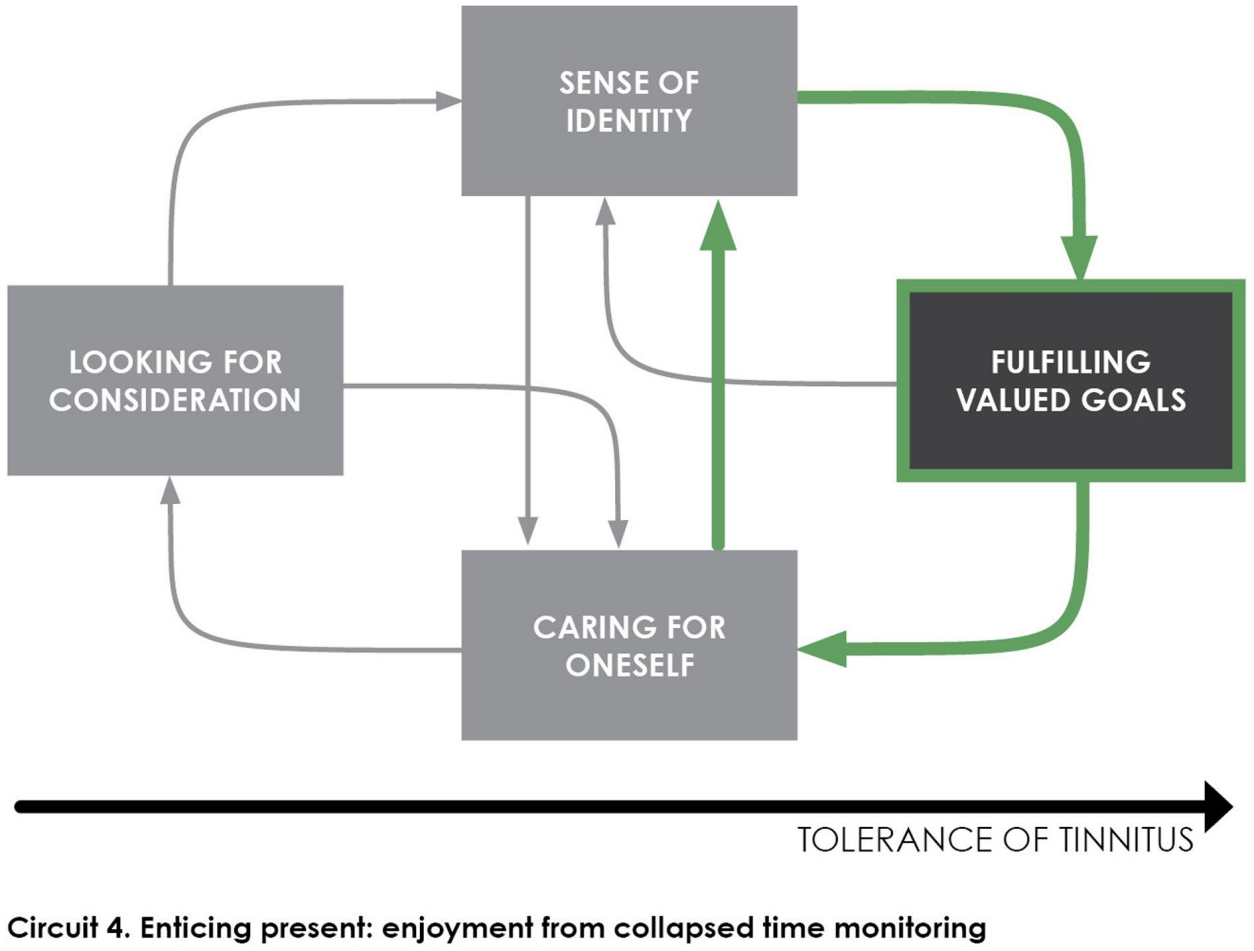
The individual develops long-term self-confidence immersing oneself in the present moment, by undertaking intrinsically rewarding activities and disregarding the presence of tinnitus. A virtuous circle sets up, with enriched experiences providing broader perspective over the monotony of tinnitus, as the individual can retrospectively contrast it with longer tinnitus-free situations.

Building on the role of frustration in annoyance variability, the Tolerance model posits that *fulfilling valued goals* strengthens the individuals’ sense of identity and contributes to growing self-confidence in living with tinnitus (Dauman et al., 2023). As depicted in Figure 5, patients get involved in a virtuous circle from the time they manage to fulfill personal goals, taking care for themselves as well as others with whom they share meaningful activities. The relation between engagement in meaningful activities and their self-confidence essentially depends on their perception of time.

Many patients manage to put tinnitus in the background of awareness when they are engrossed in activities they have freely chosen and are able to carry out without any constraint (e.g., Adams et al., 2010). Individuals have *intrinsic* motivation for choosing these activities (Slater and Terry, 1987), i.e., they enjoy performing them without caring about tinnitus. Manual work, gardening, talking with friends or playing sport, are the most usual activities that patients report as being associated with self-induced relief (Dauman and Dauman, 2021), even though the latter is usually momentary. Also well known is the detrimental influence of awareness of time on intrinsic motivation (Conti, 2001). Thinking about time disrupts one’s interest for and willingness to be absorbed in activities, and highly motivated individuals rarely focus on the passage of time (*ibid.*). In fact, persistency in goal-directed behavior is a selective process that abates from awareness irrelevant stimuli (Gable and Poole, 2012) and gives monopoly to the monitoring of one’s current progress towards goal acquisition (Threadgill and Gable, 2018). *Self-scrutiny* is the most impeding obstacle to enjoyment in carrying on one’s activities (Nakamura and Csikszentmihalyi, 2009), and the fading of self-awareness is a hallmark feature of complete engagement with rewarding activities (i.e., optimal experience or ‘flow’ see Csikszentmihalyi, 1990). When the individual enters in a flow state, both the notions of self and time lose their negative influence on pursuing rewarding goals.

Paradoxically, the main way for patients to gain long-term self-confidence is to engage actively in the present moment. Individuals who understand this point stop longing for immediate relief (Marks et al., 2022) and rather explore with greater attention their relation to themselves and to others (Marks et al., 2020). Acceptance of tinnitus notably changes the *quality* of experience in patients, who become more appreciative of others’ skills and endeavors, and more sensitive to the beauty of nature that surrounds them (*ibid.*). They also become more assertive in their relationships with others (Andersson and Edvinsson, 2008), taking responsibility in selecting their encounters in a more judicious way (Dauman and Dauman, 2021). As for elderly individuals who have a more limited number of years ahead of them, *time itself* becomes more valuable for tinnitus sufferers who know that their resources are restricted (see Carstensen et al., 1999). This resonates with the particular appreciation that is attached to dedicated professionals who spend time listening them and explaining tinnitus mechanisms to them (Marks et al., 2019; Munir and Pryce, 2020; Pryce et al., 2023). Experience-sharing in support group (Pryce et al., 2019) is another illustration of this reconsidered value of time spent with others whenever receptive, as is the willingness to return the attention one received by becoming a member of a patient organization (Dauman et al., 2017). Having a sense of belonging to a patient community plays a key role in the acceptance of tinnitus (Pryce et al., 2019; Marks et al., 2022), because sufferers do not feel isolated among others who cannot relate (Marks et al., 2020). The changes in patients’ attitude towards the present highlight the *time paradox* inherent in the acceptance of tinnitus. Tinnitus-induced distress chronically distracts sufferers from paying attention to their experience. The intrusion of tinnitus into their self-perception causes them to flee the present moment and engage in avoidance of tinnitus. Instead, exploring the present allows them to cultivate persisting attention to their experience. Therefore, patients realize how much of the improvement in acceptance comes through the attenuation of self-consciousness, which becomes part of a broader perception of the present. Ongoing perceptions that are simultaneous with the tinnitus (e.g., external and internal sounds, attitudes of and conversations with others) can only be integrated into sufferers’ perception by paying greater attention to these events. This process paradoxically involves losing track of time. In doing so, patients learn to make better *use* of their time through the alleviation of their constant preoccupation with the passage of time.

Because self and time are two aspects of the same dynamic (Wittmann, 2015), the time paradox applies to the process of personal growth. Many paradigms in psychology define personal growth as a process of extending initially self-centered interests to broader social structures of which the individual is a part (James, 1890; Csikszentmihalyi, 1990; Antonovsky, 1996; Ryan and Deci, 2000; Delle Fave and Massimini, 2005). Such an affiliation process provides life goals for individuals who cultivate skills (Delle Fave and Massimini, 2005) and satisfy basic psychological needs (Ryan and Deci, 2000) through an extended complexity of their self. Since enjoyment is the condition for consistent goal attainment, individuals’ engagement in the present moment is the building block for lifelong goal attainment (Csikszentmihalyi, 1990). In other words, the process of personal growth is enabled by self-immersion in a wide range of activities that *transcend* the current experience of self. As with the time paradox, personal growth involves losing sight of oneself by committing to broader values and goals (*ibid.*). Because multiple stressors are likely to interfere with such goals, the ability to make sense of disorder protects individuals from health threats (Antonovsky, 1996). In fact, individuals who experience tinnitus-related distress lose a sense of purpose after the onset of tinnitus (Davis and Morgan, 2008). Those who are more accepting of tinnitus in their self-perception find resources in social structures (e.g., the health care system) allowing them to make sense of their anxiety. In the latter, a sense of pride permeates self-narratives and reflects personal growth (Erlandsson et al., 2020).

Social life that surrounds sufferers is not as much present as tinnitus in self-perception. Yet, it is worth noting that smooth interactions and shared enjoyment distract individuals from self-scrutiny (Deinzer et al., 2017). Furthermore, enriched social situations (e.g., dance performance) provide individuals with experiental changes that they associate restrospectively with extended duration of pleasant time (*ibid.*). Such restrospective judgment of time would be instrumental in building self-confidence in acceptance of tinnitus. In particular, this may help individuals to qualify their sense that tinnitus is omnipresent (see Marks et al., 2020). Enjoying the moment with others is the hallmark feature of an *ego-moving perspective*, without being self-centered. The lively presence of others gives the individual a sense of being part of the same moment as them, which is essential in the acceptance of tinnitus over time.

## 5. Discussion

The major finding of the present analysis is the psychological connection we identified between the *heterogeneity* of time perception and the *time paradox*. As decisively distinguished by James (1890), the perception of our future and our past is symbolic, whereas our perception of recent past and present moment is intuitive. Human perception being essentially future-oriented, at every moment of our perceptual life there is a tension between the (symbolic) anticipation of goal and the (intuitive) perception of the progress towards this goal. The time paradox originates from this tension. One cannot be fully *committed* to a given activity and, simultaneously, anticipate the changes one wants to see *happen*.

The most compelling desire of patients with tinnitus is that they no longer perceive tinnitus in their awareness field (Pryce and Chilvers, 2018). The problem is that exaggerated focus on this wish only leads to increased time- and self-awareness, with a sense of growing frustration from being unable to get rid of tinnitus (Dauman and Dauman, 2021). On the contrary, individuals’ relief from the intrusiveness of tinnitus requires *the acceptance of the time paradox.* One can only achieve the self-defined goal by forgetting about that goal and being fully devoted to the accomplishment of the corresponding act. Indeed, patients who better admit tinnitus in their self-perception are those who soften their struggle against it (*ibid.*). Instead, they dedicate themselves more exclusively to each moment of their lives and to those around them (Marks et al., 2020). They get closer to their goal by avoiding to put all their thoughts into it.

The psycho-social perspective of the Tolerance model leads to emphasize distinct components in the theoretical account of patients’ experience in comparison with other tinnitus models. For instance, the need for consideration from others and the search for valued goals, which provide a sense of fulfillment in individuals despite the presence of tinnitus, play no specific role in the Neurophysiologic model (Jastreboff and Hazell, 2004). The model is based on animal behavior that shows similar patterns with human behavior, such as the need for self-preservation in the face of stressors that tax resources in the individual (i.e., the variable ‘caring for oneself’ in the present model). Yet, animal behavior hardly provides clear information about how social recognition (i.e., sense of belonging) may interact with the intrusiveness of tinnitus in self-perception. In human, verbal data (i.e., interviews and questionnaires) consistently show how important is *others’* behavior in the experience of tinnitus sufferers. In fact, perceived lack of understanding from interlocutors or dedicated attention to sufferers result in opposite outcomes regarding their sense of ability to cope with tinnitus (see, e.g., Marks et al., 2022).

Moreover, human behavior is characterized by individuals having *perspective* on their time, constantly appraising their present behavior in regard to future, long-term outcomes (Zimbardo and Boyd, 1999). Tolerance model alines with this hallmark feature of human perception of time (Wittmann, 2017), by emphasizing the role of goal-directed behavior in patients’ willingness to accept tinnitus over time (i.e., the fulfillment of valued goals). To our knowledge, no specific role is attributed to time perspective in the Neurophysiological model, an observation which is consistent with animal behavior that is more restricted to immediate perception of changes in the environment. Putting foreward conditioning processes (i.e., automatic and subconscious) between the tinnitus signal and negative emotions, the model would place less emphasis on cognitive processes than do psychological models (McKenna, 2004). Further exploration on the potential role of time perception in the Neurophysiologic model (e.g., through the importance of counseling, in addition to sound therapy) would be of interest in the future.

Tolerance model share common features with other psychological models of tinnitus that have been proposed in the literature. In particular, these models all hypothesize *self-perpetuating processes* that maintain tinnitus-induced distress over time. The Cognitive-behavioral model of tinnitus proposed by McKenna et al. (2014) emphasizes the role of negative thoughts about tinnitus that fuel feedback loops inducing greater autonomic arousal, selective attention and repetitive monitoring of tinnitus, in addition to safety behaviors in sufferers who aim to escape from intrusiveness (e.g., avoiding silence and impulsive alcohol/drugs consumption). Similarly, the Fear-avoidance model proposed by Cima et al. (2019) (see also Cima et al., 2011) suggests that fear of tinnitus leads to hypervigilance towards its presence and search for short-term reliefs through distraction and avoidance of situations that increase salience (e.g., quiet environments and stressful social situations). The same model further suggests that such strategies turn out to be unhelpful in the long term, because avoidance exacerbates sensitivity to tinnitus and, ultimately, social withdrawal that becomes necessary in order to keep tinnitus under control. Eventually, the Neurocognitive model that is supported by Trevis et al. (2016) argues that the salience of tinnitus results from a functional imbalance in the interaction of the cognitive control network (CCN) which is directed towards specific aims to be achieved in the environment, and the autobiographical memory network (AMN) whose neural activity translates into self-focus thinking and mind-wandering. The model posits that salience of tinnitus is exacerbated by the joint action of hypoactivity in the CCN (i.e., lack of consistency in individuals’ goal-directed attention) and hyperactivity in the AMN (i.e., ruminations about the interference of tinnitus with life expectations).

In regard to time perception, the Cognitive-behavioral model (McKenna et al., 2014) and the Fear-avoidance model (Cima et al., 2019) would both emphasize the detrimental role of patients’ symbolic sense of time in tinnitus-induced distress (i.e., how they mentally represent their past ‘without tinnitus’ and their future associated with its ‘unlimited’ presence). The Neurocognitive model (Trevis et al., 2016) would specifically illuminate patients’ intuitive sense of time (i.e., their recent past associated with changes in the interplay between self-perception and their surrounding). In particular, the model focuses on how interference becomes more salient with the interruption of goal-directed behavior and is associated with rumination about the loss of distraction from self-awareness. We would suggest that the Tolerance model contributes to psychological research in tinnitus by integrating patients’ experience of enticing moments, that are associated with the attenuation of time- and self-awareness, into a coherent framework of time perception. The model broadens the current scope on behaviors in patients by recognizing the essential role of rewarding activities that induce a flow state in the acceptance of tinnitus. Moreover, the consideration for patients’ perception of time in relation to their self-confidence allows for new perspectives on the heterogeneity in the severity of tinnitus (Cederroth et al., 2019). In particular, the contrast of individual experiences that are dominated by a time-moving perspective (i.e., anxiety and depression) and those that show an ego-moving perspective (i.e., embodiment and acceptance of tinnitus) may provide a novel avenue towards psychological resources and personal growth in individuals with chronic tinnitus.

**Figure 6:**
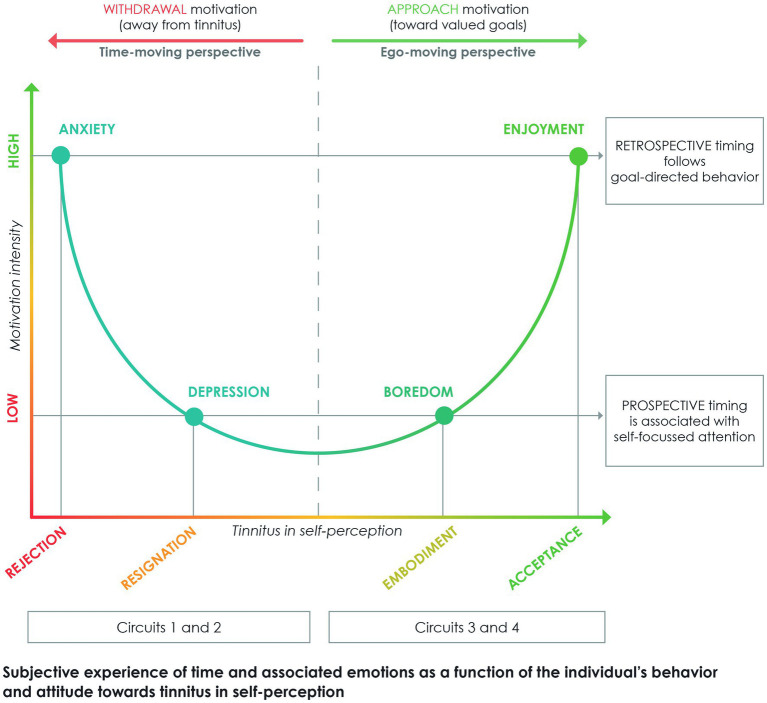
Psychological framework for future research about changes in time perception in patients with chronic tinnitus. The ordinate axis distinguishes high and low motivation in the individual’s behavior, either towards avoidance of tinnitus (i.e., withdrawal motivation) or pursuit of valued goals (i.e., approach motivation). The abscissa axis indicates the individual’s attitude towards the presence of tinnitus in self-perception (i.e., rejection, resignation, embodiment, and acceptance). In the patient’s emotional experience, anxiety and depression are associated with a time-moving perspective (i.e., individual stationary, tinnitus in the front of awareness), whereas boredom and enjoyment are associated with an ego-moving perspective (i.e., individual in motion, tinnitus stationary). Relation of these coordinates to the time paradox are explained in the text.

Building on the heterogeneity of time perception and the time paradox, we will now provide a psychological framework (Figure 6) for future research about acceptance for patients with chronic tinnitus. The figure is organized around a vertical dashed line that separates, one from the other, behaviors and associated emotions characterizing an individual’s *rejection* of the time paradox (left side of the figure) and the *acceptance* of this paradox (right side). Although this framework provides hypotheses for inter-individuals comparisons (see below), it was designed in priority to more closely explore intra-individual changes in tinnitus perception over time.

An individual who rejects the time paradox wants to be *immediately* relieved from tinnitus. Experiencing the persistence of tinnitus, the sufferer is dominated by a *time-moving perspective*. Tinnitus returns systematically in the forefront of awareness whatever the individual undertakes to avoid it (Hesser et al., 2009), a finding that throws them into a stationary position associated with helplessness (Richmond et al., 2012). Persistent loss of agency in the sufferer results in high levels of anxiety and depression, which both are central to tinnitus-related distress (Hesser and Andersson, 2009; Langguth et al., 2011), and relate to attitudes of *rejection* and *resignation* towards tinnitus in self-perception, respectively, (see also Marks et al., 2020).

Tolerance model suggests that anxiety and depression are related one to another in regards to the individual’s disengagement from unattainable goals (see Nesse, 2000; Wrosch et al., 2003). Endless pursuit of avoidance of tinnitus leads the sufferer to exhaustion, which is a waste of energy that runs counter to self-preservation (Höbfoll, 1989). The present analysis suggests that high levels of anxiety reflect constant expectations and fear towards the future (Cima et al., 2011), while high levels of depression indicate continuous rumination towards the past (Erlandsson et al., 2020). Hence, the alternation between one’s rejection (i.e., anxiety-driven) and resignation (i.e., depression-driven) in front of tinnitus essentially characterizes individuals’ *inattention to the present moment*. This view on time perception is in line with a mindfulness-based approach to tinnitus (McKenna et al., 2018) and highlights the notion of ‘distorted perception’ of tinnitus that is promoted in the Cognitive-behavioral model (McKenna et al., 2014). A time-moving perspective (McGlone and Pfiester, 2009; Richmond et al., 2012) accounts for tinnitus-related distress in the individual who find themselves stuck in a stationary position, unable to *move forwards* due to the omnipresent intrusiveness of tinnitus (see Ruscher, 2011).

Acceptance of the time paradox fundamentally involves an *ego-moving perspective* towards one’s future and valued goals to come (see Boroditsky, 2000; McGlone and Pfiester, 2009). An individual with tinnitus who accepts the time paradox typically focuses greater attention on the present moment (Marks et al., 2020) and spares one’s resources in adopting a regular lifestyle (Dauman and Dauman, 2021). The struggle against tinnitus is replaced by wiser attitudes towards its presence in self-perception, i.e., *embodiment* (Munir and Pryce, 2020) and *acceptance* (Hesser et al., 2015). As pointed out by Munir and Pryce (2020), the presence of tinnitus in self-perception fuels a feeling of dissociation between body and self, from which the individual tries to escape with the help of chosen auditory environment (e.g., sound generators, music, conversations). The individual also relies on routines (e.g., walking outdoors, doing gymnastics) to shorten the duration of time and overcome boredom induced by the dull presence of tinnitus (Avni-Babad and Ritov, 2003; Elpidorou, 2014). Because chronic tinnitus spoils sufferers’ experience with their life, immersing into rewarding social activities helps them to distract from adversive self-awareness (Moynihan et al., 2021). Thus, an ego-moving perspective over tinnitus involves the alternated experiences of boredom and enjoyment of the moment with others. Serenely allowing both to be part of self-perception—boredom as much as enjoyment—is essential to the acceptance of tinnitus (McKenna et al., 2018; Marks et al., 2020).

Individuals’ willingness to engage in the present moment relies on their acceptance of the time paradox. The benefit for patients with tinnitus is twofold. Finding intrinsic reward allows them to lose track of time and self-awareness at once. In addition, meaningful experiences also have greater contextual changes that, retrospectively, are perceived with greater length in one’s memory (Block et al., 2010; Wittmann, 2015; Deinzer et al., 2017). In fact, engaging in the moment is the *main* way by which individuals experience gradual acceptance of tinnitus in their self-perception. Associated with greater contextual changes, multiple memories of tinnitus-free situations help them qualify their belief about the omnipresence of tinnitus. This view is consistent with studies on autobiographical memories in chronic pain patients, where impoverished memories were reported to be associated with greater intrusiveness (Meyer et al., 2015; Quenstedt et al., 2021). Coupled with an increased self- and time awareness, prospective timing (Zakay, 2014) can only confirm to the individual their experience of an unlimited presence of tinnitus. Therefore, an ego-moving perspective over tinnitus provides the individual with self-induced relief *mediated by* one’s engagement with *the moment*.

The theoretical consistency of this framework allows for general hypotheses that require further experimental testing with respect to the time paradox. Inter-individual comparisons may be undertaken from the cross-sectional survey by Beukes et al. (2022), which distinguished four clusters of patients with associated levels of annoyance severity: *debilitating* tinnitus (cluster 1), *distressing* tinnitus (cluster 2), *annoying* tinnitus (cluster 3) and *accepting* tinnitus (cluster 4). The following predictions can be made for future psychological research, attempting to match each cluster with a distinct attitude towards tinnitus in self-perception: rejection (cluster 1), resignation (cluster 2), embodiment (cluster 3) and acceptance (cluster 4).

1. A *time-moving perspective* will be characteristic of individuals’ perception in clusters 1 and 2, in contrast with an *ego-moving perspective* that defines individuals’ perception in cluster 3 and 4. In the framework, this hypothesis contrasts individuals who reject the time paradox (clusters 1 and 2) from those who accept it more widely (clusters 3 and 4). This hypothesis can be tested by experiments on temporal agency assignment (see McGlone and Pfiester, 2009; Richmond et al., 2012) and by eye-tracking experiments (see Pfaltz et al., 2021).
2. In real *waiting situation*, individuals in cluster 1 and 2 will show more impulsivity, associated with an *overestimation* of duration and perceived *slower* passage of time, as compared with individuals in clusters 3 and 4. This hypothesis builds on the role of impulsive behaviors to escape from adversive self-awareness and self-focused attention. This hypothesis can be tested by questionnaires (see Jokic et al., 2018).
3. *Autobiographical memory* will be more *specific* (i.e., contextualized) in individuals in cluster 3 and 4, as compared with individuals in cluster 1 and 2. This hypothesis builds on the role of engagement in the moment, which provides individuals with enriched contextual changes that are associated with enhanced memory retrieval. This hypothesis can be tested by words association (see Williams et al., 1996) or sentence completion tasks (see Quenstedt et al., 2021), providing further data to the study by Andersson et al. (2003).
4. *Time perspective* will be *broader* (i.e., with long-term goals) for individuals in groups 3 and 4, compared to individuals in groups 1 and 2. This hypothesis builds on the time paradox, according to which deeper attention to the present, associated with lucidity about one’s opportunity for action, expands individuals’ ability to schedule future goals (see Zimbardo and Boyd, 2008). The accuracy of this hypothesis can be tested by a specific questionnaire (the ZTPI, Zimbardo Time Perspective Inventory, see Zimbardo and Boyd (1999)).

## 6. Conclusion

This theoretical analysis on time perception in tinnitus patients contributes to the current knowledge about the temporal aspects that lead to distinguish acute and chronic tinnitus from different clinical and basic research perspectives (Tunkel et al., 2014; de Ridder et al., 2021; Mazurek et al., 2022). The preceding remarks suggest that metric assessment of tinnitus (i.e., duration since onset) can be advantageously completed by further research into how patients *spend* their time and how they perceive changes in their self-perception induced by their *own behavior*. In particular, individuals who take a time-moving, shorter perspective on their future would be more likely to be sensitive to tinnitus-induced heightened awareness of self that comes with a greater awareness of the passage of time. In these patients, the increased attention to time and self would contribute to more frequently interrupted behavior and impaired attention to the present moment that is essential for growing acceptance. Thereby, exploring time perception in patients with recent onset of tinnitus (e.g., through questionnaires) may provide further information about the likelihood of spontaneous improvement in their reaction to the symptom (see Tunkel et al., 2014). The study of time perception in patients with tinnitus also echoes with contemporary reflection in psychology about the sense of agency in individuals under stressful situations (Swann and Jetten, 2017). In line with an increased attention to the professionals’ discourses and others’ attitudes towards patients (Pryce et al., 2023), the present analysis contends that time perception is a *social* perception. This inquiry also led us to articulate one to another several psychological impacts of tinnitus that are usually considered as being separated co-morbidities. In particular, anxiety and depression are viewed as two facets of individuals’ struggle towards an unattainable goal (i.e., tinnitus suppression). Further research is needed to investigate the hypothesized role of depression (Nesse, 2000) in regard to self-preservation (i.e., suicide risks) and resources conservation in patients with tinnitus (i.e., long-term stress coping). In agreement with acceptance-based approaches, the study of time perception also led us to broaden a pathogenic view on tinnitus (i.e., focusing on detrimental factors) by integrating positive factors that modulate time awareness, such as enjoyment in rewarding activities (Csikszentmihalyi, 1990). This exploration provided further support to the role of frustration and goal-fulfillment in chronic tinnitus (Dauman and Dauman, 2021). We contend that future research on self-awareness is a promising avenue to further exploring moment-to-moment annoyance variability. In particular, the connection between enjoyment (i.e., dissipated self-awareness) and personal growth should be further investigated. Mindfulness-based approaches are especially designed to address this goal (McKenna et al., 2018). Research on personal growth in relation to time perception will also benefit from narrative-based approaches in the field of psychotherapy (Erlandsson et al., 2020). Eventually, the connection between the heterogeneity of temporal perception and the time paradox may provide practical insights to suffering patients and health professionals who dedicate time to help them.

## Data availability statement

The original contributions presented in the study are included in the article/supplementary material, further inquiries can be directed to the corresponding author.

## Author contributions

The author confirms being the sole contributor of this work and has approved it for publication.

## Funding

This study was supported by France Acouphènes (French Tinnitus Association), the MSHS and the CAPS composante URm RPPSY - 15297.

## Conflict of interest

The author declares that the research was conducted in the absence of any commercial or financial relationships that could be construed as a potential conflict of interest.

## Publisher’s note

All claims expressed in this article are solely those of the authors and do not necessarily represent those of their affiliated organizations, or those of the publisher, the editors and the reviewers. Any product that may be evaluated in this article, or claim that may be made by its manufacturer, is not guaranteed or endorsed by the publisher.
